# Miscellaneous Items

**Published:** 1890-02

**Authors:** 


Chilling the Feet and its Consequences.—Dr. Munde says that to the imprudent act of getting out of bed without protecting the feet—one so commonly committed by women without thought of the consequences—may be traced many an attack of cellulitis brought on by the sudden though momentary exposure of the feet to cold. It has caused more diseases to women previously healthy than could result from any other single act of imprudence.—*Med. Standard.*

Stuttering.—It is a well-known fact that stutterers, when speaking in a whispering voice, show no impediment of speech. A new method of treatment has been advocated by Dr. Coen and is as follows: In the first ten days speaking is prohibited. This will allow rest to the voice, and constitutes the preliminary state of treatment. During the next ten days speaking is permissible in the whispering voice, and in the course of the next fifteen days the ordinary conversational tone may be gradually employed.—*Kansas Med. Jour.*, Oct., 1889.

WATER for drinking purposes should never be below fifty degrees. We can almost always get it, even in the hottest weather, as cool as this by letting it run for a minute or two from any household faucet or drawing it from any country well. I am quite sure that if ice water should generally be discarded as a drink, the average duration of life would be lengthened and existence rendered more tolerable.—*William H. Hammond.*

ACTING upon suggestions in a communication signed by Drs. S. Weir Mitchell, D. Hayes Agnew, William Pepper, J. W. DaCosta, W. W. Keen, H. C. Wood, and William Hunt, the Board of Health of Philadelphia, on November 29, adopted a resolution in which it "strongly advises the boiling of all drinking water and milk at least twenty minutes, as the simplest and best means of purification."

APPLES FOR BRAIN WORKERS.—Apples contain a larger amount of phosphorus, or brain food, than any other frnit or vegetable, and on this account they are very important to sedentary men, who work their brains rather than their muscles. They also contain the acids which are needed every day, especially for sedentary men, the action of whose liver is sluggish, to eliminate the effete matter, which, if retained in the system, produces inaction of the brain, and, indeed, of the whole system, causing jaundice, sleepiness, scurvy, and troublesome diseases of the skin. —*Food.*

TRANSMISSION OF DISEASE BY BRUSHES AND DENTAL INSTRUMENTS.—A discussion recently took place at the Conseil d'Hygene concerning the transmission of certain diseases by hairdressers and dentists, the brushes and instruments being used in common for all their clients. M. Lancereauz wished to have stringent measures enforced, and cited a case of phthisis which Dr. Cochrane, an American dentist, alleged was transmitted by a dentist's instrument. M. Dujardin-Beaumetz and others declared that there were great difficulties in the way, but recommended great care in schools and public institutions.

